# Cancer-related alopecia and wig acquisition: how age, sex, and treatment affect patient choices

**DOI:** 10.1007/s00520-025-09318-8

**Published:** 2025-03-15

**Authors:** Hideki Katayama, Eiki Ichihara, Ayako Morita, Go Makimoto, Shunsuke Kagawa, Ayano Ishii, Masahiro Tabata, Yoshinobu Maeda

**Affiliations:** 1 Department of Palliative and Supportive Care, Okayama University Hospital, Okayama, Japan; 2 Center for Clinical Oncology, Okayama University Hospital, Okayama, Japan; 3 Department of Allergy and Respiratory Medicine , Okayama University Hospital, Okayama, Japan; 4 Integrated Support Center for Patients and Self-Learning , Okayama University Hospital, Okayama, Japan; 5 Department of Hematology and Oncology, Okayama University Hospital, Okayama, Japan

**Keywords:** Cancer, Alopecia, Wig purchases, Appearance care, Patient support

## Abstract

**Purpose:**

This study aimed to explore the prevalence and cost of wig purchases among patients with cancer in Okayama Prefecture, Japan, and examine the relationship between wig purchases and various demographic, social, and clinical factors. The findings aim to provide insights into appearance care and support systems for patients with cancer, particularly wig subsidies.

**Methods:**

A survey was conducted between July and August 2023 among 3000 patients with cancer at 13 designated cancer care hospitals in Okayama Prefecture. Data on demographics, cancer treatment status, and wig purchase details were collected. Statistical analyses, including the Mann–Whitney *U* test, chi-square test, and logistic regression, were performed to identify factors significantly associated with wig purchases.

**Results:**

Among the 863 respondents, 31.4% (271 patients) reported purchasing wigs. Factors significantly associated with wig purchase included young age (odds ratio [OR] = 1.04), female sex (OR = 1.61), and current cancer treatment (OR = 1.16). No significant correlation was found between wig purchase and household income, although higher-income patients tended to purchase more expensive wigs.

**Conclusion:**

The findings suggest that younger female patients with cancer and those undergoing treatment were more likely to purchase wigs, highlighting the importance of appearance care and the need for enhanced financial support for low-income patients.

## Introduction

Patients with cancer undergoing treatment often experience various types of pain and disabilities [1]. Among these, alopecia (hair loss) is a common side effect of cancer treatment, affecting approximately 65% of the patients undergoing chemotherapy [2, 3]. While hair loss is usually temporary, it can sometimes lead to permanent baldness [4, 5]. Particularly, radiation therapy-induced hair loss may result in permanent baldness, depending on the area being treated [6]. Alopecia causes significant psychological distress, especially among females [7, 8]. Hair is an important aspect of self-image for many females, and its loss can lead to reduced self-esteem, depression, and anxiety [9]. In some cases, this psychological burden can exceed that of a mastectomy [10]. Additionally, alopecia may affect social behavior, prompting concealment, social support avoidance, isolation, and information-seeking behaviors [7]. Concerns about hair loss can lead patients to refuse effective treatments, affecting their treatment decisions [3, 11]. While temporary alopecia can have lasting effects, these psychological changes are especially pronounced in patients with permanent hair loss [12]. Social expectations regarding appearance, especially in Asian cultures, are high, and the impact of hair loss may be more severe in these regions [13]. Furthermore, appearance and supportive care have become global issues. Despite the importance of supportive care in cancer treatment, many low- and middle-income countries face funding shortages.

Although awareness of the effects of cancer treatment-induced alopecia is growing, healthcare professionals, especially physicians, often underestimate its significance as it is not a life-threatening side effect [14]. Hair loss management is often neglected as the primary focus is overall health and treatment efficacy. Moreover, alopecia can exacerbate other chemotherapy side effects, further diminishing patients’ quality of life [7]. For example, when physical side effects such as fatigue or nausea occur, the additional psychological stress from hair loss can further decrease patients’ motivation to continue treatment [10, 15]. Studies have highlighted the importance of addressing body image concerns through psychological interventions; for instance, Sebri et al. and Fabi et al. demonstrated the positive impact of psychological interventions on emotional resilience and quality of life [16, 17].

Therefore, comprehensive management of cancer treatment-induced hair loss is essential for improving patients’ quality of life. Improving appearance can enhance patients’ well-being and reduce cancer-related stigma [18]. Various strategies to address alopecia have been considered, with recent developments focused on preventing or reducing chemotherapy-induced hair loss [4, 19, 20]. For example, scalp cooling has been shown to reduce hair loss during chemotherapy, with Rugo et al. reporting that cooling caps prevented hair loss in 66.3% of the patients [21]. However, this method is unsuitable for all patients owing to side effects or practical difficulties [22].

In this context, wigs remain popular owing to their ease of use [8, 23]. Wigs provide an immediate and visible solution that helps patients restore their self-image, communicate with family, and maintain social confidence. However, some patients struggle to find suitable wigs, and the high cost can increase the financial strain on families [24]. Additionally, information on wigs is often lacking, making it challenging for patients in rural areas to find appropriate options.

Therefore, providing financial support for wig purchases to help patients cope with chemotherapy-induced alopecia could significantly aid them psychologically [25]. In Japan, comprehensive support for patients with cancer, emphasized in the National Cancer Control Plan, highlights the importance of appearance care. While some regions offer wig subsidy programs, eligibility and subsidy amounts vary, leaving some patients without adequate support. Most previous studies have focused on urban patients or females [26, 27], leaving gaps in our understanding of the experiences of those in rural areas and other demographics.

This study aims to clarify trends in wig purchases among cancer patients and examine the factors influencing accessibility. By identifying financial, informational, and reginal barriers, we seek to enhance the awareness of appearance-related supportive care and contribute to policy improvements.

## Materials and methods

### Participant recruitment and selection criteria

This study aimed to investigate trends in wig purchases and associated factors among patients with cancer living in Okayama Prefecture, Japan. Between July and August 2023, a cross-sectional anonymous survey was conducted among patients with cancer who were receiving or who had previously received treatment at 13 designated cancer care hospitals in Okayama Prefecture (listed in the acknowledgments). These hospitals were selected according to their designation in national and prefectural cancer care networks. The survey was also distributed to patients affiliated with cancer support groups participating in the Okayama Prefecture Cancer Care Coordination Council. Participants were recruited via direct distribution of the survey within hospitals, QR code linking to the online survey, and email outreach through cancer support organizations. Responses were collected via a web-based form or by mail.

### Survey components

The survey included questions on sociodemographic (age, sex, highest education level, marital status, household head status, presence of cohabiting family members, employment status, and current household income), cancer-related (cancer type, current treatment status, and whether cancer treatment affected families’ lives or treatment choices), and wig-related (whether they had purchased a wig and its cost). Personal income and wig costs were reported in Japanese yen, with an exchange rate of US$1 = JPY¥145.54 (as of August 31, 2023).

### Statistical analysis

The relationship between wig purchase and various demographic and clinical factors was analyzed using SPSS version 29 (IBM Corp., NY, USA). The Mann–Whitney *U* and chi-squared tests were used to evaluate differences between categorical variables, with statistical significance set at *p* < 0.05. For factors found significant in univariate analysis, a logistic regression model was constructed for multivariate analysis, excluding variables with Pearson correlation coefficients > 0.5 to avoid collinearity. Spearman’s rank correlation (*ρ*) was used to assess associations between ordinal variables.

### Ethical considerations

The study was conducted in accordance with the Declaration of Helsinki and was approved by the Clinical Research Review Expert Committee of Okayama University (October 20, 2023/approval no. 2311–007). Patient consent was obtained through an opt-out procedure, with the study information provided on the hospital’s website. Personal information was anonymized and securely managed.

## Results

A total of 3000 surveys were distributed, yielding 863 valid responses regarding wig purchase. Among the respondents, 31.4% (271 individuals) reported purchasing wigs. Table 1 shows the distribution of cancer sites, which aligns with the incidence rates of major cancer types in Japan [28], indicating that the survey reflects the population of patients with cancer.

**Table 1:** Primary cancer sites

Cancer type^†^		*N*
	Breast	188
	Lung	115
	Digestive tract	194
	Liver/bile duct	35
	Pancreas	44
	Gynecologic	62
	Urogenital	68
	Head and neck	50
	Blood	61
	Others	45
	Missing	6

^†^Multiple answers

Table 2 summarizes the patients’ sociodemographic and clinical characteristics and their relationship with wig purchase. The factors of age (*r* =  − 0.29, *p* < 0.001), sex (*χ*^2^(1) = 326.3, *p* < 0.001), highest education level (*χ*^2^(1) = 18.2, *p* < 0.001), marital status (*χ*^2^(1) = 12.8, *p* < 0.001), household head status (*χ*^2^(1) = 161.7, *p* < 0.001), presence of a cohabiting partner (*χ*^2^(1) = 9.21, *p* = 0.003), and treatment status (*χ*^2^(1) = 17.2, *p* < 0.001) were significantly associated with wig purchases. In contrast, no significant association was found with the presence of cohabiting family members (*χ*^2^(1) = 0.39, *p* = 0.61), employment status (*χ*^2^(1) = 0.34, *p* = 0.59), household income (*p* = 0.076, *r* = 0.062), or the impact of cancer on daily life and treatment (*χ*^2^(1) = 2.75, *p* = 0.097).

**Table 2:** Patients sociodemographic characteristics and univariate analysis results

		Bought a wig		Did not buy		
		*N*	(%)	*N*	(%)	*p* value
Current age group						*p* < 0.001*
	20 s	1	(0.4)	1	(0.2)	
	30 s	12	(4.4)	7	(2.2)	
	40 s	48	(17.7)	27	(8.8)	
	50 s	76	(28.0)	107	(21.4)	
	60 s	73	(26.9)	176	(29.1)	
	70 s	54	(19.9)	220	(32.0)	
	80 s	7	(2.6)	47	(6.3)	
	90 s	0	(0)	1	(0.1)	
Sex						*p* < 0.001
	Female	262	(97.0)	182	(30.7)	
	Male	8	(3.0)	410	(69.3)	
Education levels achieved						*p* < 0.001
	Higher than a high school graduate	157	(58.4)	250	(42.7)	
	High school graduate or lower	112	(41.6)	336	(57.3)	
Marital status						*p* < 0.001
	Spouse/Partner	180	(66.4)	461	(77.9)	
	No	91	(33.6)	131	(22.1)	
Primary householder						*p* < 0.001
	Yes	85	(31.5)	454	(76.7)	
	No	185	(68.5)	138	(23.3)	
Living with partner						*p* = 0.002
	Yes	184	(70.5)	457	(80.0)	
	No	77	(29.5)	114	(20.0)	
Living arrangement						*p* = 0.534
	Cohabitation	246	(94.3)	544	(95.3)	
	Living alone	15	(5.8)	27	(4.7)	
Current treatment status						*p* < 0.001
	Under treatment	233	(86.3)	435	(73.6)	
	End of treatment	37	(13.7)	156	(26.4)	
Current job status						*p* = 0.559
	Working	136	(55.7)	291	(53.5)	
	without an occupation	108	(44.3)	253	(46.5)	
Annual household income (yen)						*p* = 0.076*
	< 1,000,000	17	(6.6)	45	(8.0)	
	1,000,000 ≤ 3,000,000	78	(30.1)	190	(33.9)	
	3,000,000 ≤ 5,000,000	73	(28.2)	159	(28.3)	
	5,000,000 ≤ 7,000,000	36	(13.9)	77	(13.7)	
	7,000,000 ≤ 9,000,000	28	(10.8)	40	(7.1)	
	9,000,000 ≤ 11,000,000	12	(4.6)	24	(4.3)	
	> 11,000,000	15	(5.8)	26	(4.6)	
Impact on treatment and daily life						*p* = 0.097
	Yes	123	(45.9)	234	(39.9)	
	No	145	(54.1)	353	(60.1)	

*Mann–Whitney *U* test

Among the significantly associated variables, household head status and the presence of a cohabiting partner showed strong collinearity with sex (correlation coefficient, 0.66) and marital status (correlation coefficient, 0.83); therefore, these variables were excluded from the multivariate analysis. The remaining factors with significant results in the univariate analysis were included in the logistic regression analysis. The multivariate analysis revealed that younger age (OR = 1.04, 95% CI [1.02, 1.05]), female sex (OR = 1.61, 95% CI [1.48, 1.74]), and currently undergoing treatment (OR = 1.16, 95% CI [1.10, 1.22]) were significantly associated with wig purchase (Table 3). These findings are consistent with those of previous studies showing that younger females undergoing treatment tend to be more concerned about changes in their appearance [7].

**Table 3:** Results of logistic regression analysis of relationships between wig purchasing behavior and various factors

	OR	*p* value
Current age group	0.96	*p* < 0.001
Sex	1.61	*p* < 0.001
Education levels achieved	1.06	*p* = 0.148
Marital status	1.00	*p* = 0.938
Current treatment status	1.16	*p* < 0.001

*OR* odds ratio

**Fig. 1:**
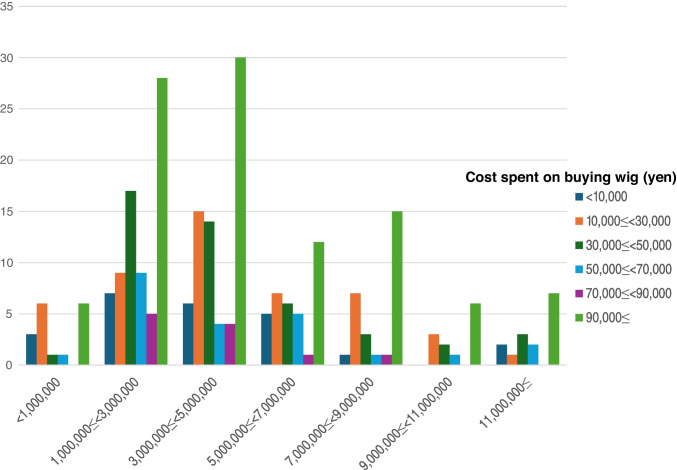
Annual household income and wig purchase costs

Figure 1 shows the relationship between wig purchase costs, household income, and impact on daily life. While there are differences in product quality, brand, and retailer, in Japan, fashion wigs can be purchased for several thousand yen to approximately ¥30,000, medical wigs made of synthetic hair cost approximately ¥50,000, and medical wigs made of mixed fibers cost approximately ¥100,000. Although there was a trend toward higher household income correlating with higher wig purchase costs, no statistically significant association was found (*ρ* = 0.062, *p* = 0.325, *N* = 256).

## Discussion

This study identified young age, female sex, and current treatment as essential factors associated with wig purchase among patients with cancer. These findings are consistent with previous studies highlighting the psychological and social importance of appearance, particularly for young female patients and those undergoing treatment [3, 7, 9, 29]. Hair loss affects more than just appearance; it significantly impacts body image, self-perception, and psychological well-being, often leading to reduced self-esteem, social withdrawal, and emotional distress [10]. Thus, patients experiencing alopecia require comprehensive supportive care, including financial aid for wigs and psychological interventions.

This study suggests that wig purchases go beyond cosmetic concerns and are deeply tied to body image and self-identity. Hair loss, particularly for young female patients, can feel like a loss of control over one’s body, affecting self-expression and femininity [7, 8]. Maintaining a stable body image is crucial for emotional resilience, and wigs help restore a familiar self-image and social confidence [3].

Therefore, psychological interventions play a critical role in supporting cancer patients dealing with body image distress. Seri et al. emphasize that tailored psychological support can help cancer survivors navigate changes in their appearance, reinforcing emotional well-being and self-acceptance [16]. Additionally, Fabi et al. highlight that long-term psychological interventions can mitigate the distress associated with altered physical appearance, contributing to better mental health outcome [17]. These findings suggest that combining financial and psychological support for wig use could offer a more holistic approach to managing hair loss-related distress in cancer patients.

Notably, no significant correlation was observed between household income and wig purchase. Patients with higher incomes tended to purchase more expensive wigs; however, wig-buying behavior itself was not significantly influenced by income level. This point suggests that many patients with cancer, particularly females and young adults, view wigs as essential, regardless of financial constraints. These patients may prioritize maintaining their appearance and psychological health in the face of financial hardship, highlighting the importance of social and psychological pressures on health-related decisions. Previous studies have shown that maintaining appearance is crucial for self-esteem and quality of life, potentially driving patients to purchase wigs despite limited financial resources [15].

From a health policy perspective, these findings indicate the need for greater financial support for wig purchases, particularly for low-income patients. Regional subsidy programs should be expanded nationwide to ensure equitable access to appearance care for all patients, regardless of socioeconomic status. Furthermore, information and guidance on the available financial support should be a standard part of cancer treatment consultations. This approach would reduce the financial burden on patients and improve their mental health by alleviating some of the anxiety associated with hair loss.

However, this study has some limitations. First, the response rate was lower than expected, possibly leading to selection bias. Those who participated in the survey may have been more concerned about their appearance or were more likely to buy a wig, and this could have biased the results. Second, as a cross-sectional study, this study could not assess changes in wig-buying behavior over time or the long-term psychological impact of hair loss [27]. Future studies should employ a longitudinal design to better understand how cancer patients’ attitudes toward wig purchase change throughout their treatment.

Furthermore, the study relied on self-reported data, which may have introduced social desirability bias. Patients may overreport the psychological impact of wig purchase and hair loss to conform to social expectations. Incorporating objective data collection methods such as interviews and observational studies may help validate these findings in future studies. Finally, it is important to consider the regional context in which this study was conducted in, i.e., Okayama Prefecture. Differences in socioeconomic status, healthcare access, and wig subsidy availability across Japan may influence wig-buying behavior. For example, patients in rural areas may have difficulty accessing affordable wigs or may not benefit from subsidy programs, impacting the generalizability of these outcomes. Extending future research to other areas, including urban and rural areas, may provide a more comprehensive understanding of how geographical and social factors affect wig acquisition among patients with cancer.

## Conclusion

This study highlights the psychological and social importance of wigs, particularly among young females undergoing treatment. Given the potential financial burden of purchasing a wig, policymakers must consider expanding subsidy programs and providing comprehensive appearance care support for all patients with cancer. Addressing these needs would improve patients’ quality of life, reduce the stigma associated with hair loss, and ultimately contribute to more holistic cancer care. However, to validate these findings further, longitudinal studies incorporating objective data collection methods across other areas, both rural and urban, are needed.

## Data Availability

No datasets were generated or analysed during the current study.
